# 

**Published:** 1847-07-01

**Authors:** 


### CONTENTS

OF THE

### MEDICO-CHIRURGICAL REVIEW,

### JULY 1, 1847.

### REVIEWS.

I.

I. Entwickelungsgeschichte des Hunde-Eies. Von Th. L. W. Bischoff ..
II. Zusätze zur Lehre vom Baue und von den Verrichtungen der Geschlechtsorgane. Von Ernst Heinrich Weber .. .. .. .. .. .. .. ..
III. Ueber die Glandulæ Utriculares des Uterus des Menschen und ihren Antheil an der Bildung der Decidua. Von Th. Ludw. W. Bischoff .. .. ..
IV. Die Entwickelung des Menschen und des Hühnchens im Eie, &c. Von Dr. M. P. Erdl . .. .. .. .. .. .. .. .. .. .. .. .. .. .. .. 1

1. Importance of Embryology .. .. .. .. .. .. .. .. .. .. 2
2. Component Parts, &c. of the Ovum .. .. .. . .. .. .. 3
3. Mode of Impregnation of the Ovum .. .. .. .. .. .. .. 6
4. Influence of the Semen .. .. .. .. .. .. .. .. .. .. .. 7
5. Time and Seat of Conception.. .. .. .. .. .. .. .. .. .. 8
6. Office of the Germinal Vesicles .. .. .. .. .. .. .. .. 9
7. Professor Weber on the Uterine Glands.. .. .. .. .. .. .. 11
8. Structure of the Placenta in the Dog .. .. .. .. .. .. .. 13
9. Dr. Weber on the Human Decidua .. . .. .. .. .. .. .. 14
10. Professor Bischoff on the Human Decidua .. .. .. .. .. .. 15
11. Of the Decidua Reflexa .. .. .. .. .. .. .. .. .. .. .. 16
12. Professor Weber on the Vessels of the Placenta .. .. . .. .. 18
13. Circulation of the Placenta .. .. .. .. .. .. .. .. .. .. 21

II.

Traité Elémentaire et Pratique de Pathologie Interne. Par A. Grisolle, M.D.P. 22

1. Fevers .. .. .. .. .. .. .. .. .. .. .. .. .. .. .. 24
   A. Typhoid Fever .. .. .. .. .. .. .. .. .. .. .. .. .. 25
   B. Typhus .. .. .. .. .. .. .. .. .. .. .. .. .. .. 28
   C. Simple Intermittent Fever .. .. .. .. .. .. .. .. .. 31
   D. Pernicious Intermittent Fevers .. .. .. .. .. .. .. .. 32
2. Inflammation—Hæmorrhages.. .. .. .. .. .. .. .. .. .. .. 33
3. Apoplexy—Poisons .. .. .. .. .. .. .. .. .. .. .. .. .. 35
4. Lesions of Nutrition .. .. .. .. .. .. .. .. .. .. .. .. 37
5. Accidental Productions foreign to the System .. .. .. .. .. 40

III.

I. A Treatise on the Structure, Diseases, and Injuries of the Bloodvessels. By Edwards S. Crisp, M.R.C.S. .. .. .. .. .. .. .. .. .. .. ..

II. Observations on Aneurism and its Treatment by Compression. By O'Bryen Bellingham, M.D. .. .. .. .. .. .. .. .. .. .. .. .. .. 42

IV.

Recueil de Memoires de Medecine, de Chirurgie, et de Pharmacie Militaires. Vol. LXII. .. .. .. .. .. .. .. .. .. .. .. .. .. .. .. .. 49

1. Fragments from the Surgical Clinique of M. Sedillot .. .. .. .. .. 49

2. Several Cases of Congelation. By Dr. Shrimpton .. .. .. .. .. 52

3. On the early Use of Injections in Gonorrhœa. By Dr. Poullain .. 53

4. Frequency of Tænia in Algeria . .. .. .. .. .. .. .. .. .. 57

5. Report of the Venereal Patients under the care of M. Marchal (de Calvi) at the Val de Grace. By M. Souhaut .. .. .. .. .. .. 57

V.

Sullo Scorbuto Indagini, Osservazioni ed Esperienze di Carlo Novellis .. .. 61
1. Apyretic or Chronic Scorbutus .. .. .. .. .. .. .. .. .. .. 63
2. Pathology of Scorbutus .. .. .. .. .. .. .. .. .. .. .. .. .. 63
3. Acute Scorbutus, or Scorbutic Synocha .. .. .. .. .. .. .. 64
4. Complications of Scorbutus .. .. .. .. .. .. .. .. .. .. .. 65
5. Curative Measures .. .. .. .. .. .. .. .. .. .. .. .. .. .. 67

VI.

Observations on the History and Treatment of Dysentery and its Combinations. By William Harty, M.D. &c. .. .. .. .. .. .. .. .. .. .. .. .. 69
1. Simple Dysentery .. .. .. .. .. .. .. .. .. .. .. .. .. .. 72
2. Affinity between Dysentery and Rheumatism .. .. .. .. .. .. 73
3. Combinations of Dysentery with Intermittent and Remittent Fevers.. 75
4. Combination of Dysentery with Typhus .. .. .. .. .. .. .. 79
5. Infectiousness of the Typhoid Form .. .. .. .. .. .. .. .. 85

VII.

On Wounds and Injuries of the Abdomen and the Pelvis. By G. J. Guthrie, F.R.S., &c. .. .. .. .. .. .. .. .. .. .. .. .. .. .. .. .. .. 88
1. Ventral Hernia.. .. .. .. .. .. .. .. .. .. .. .. .. .. .. 90
2. Employment of Sutures .. .. .. .. .. .. .. .. .. .. .. .. .. 91
3. Management of the Protruded Parts .. .. .. .. .. .. .. .. 91
4. Wounds of the Intestine .. .. .. .. .. .. .. .. .. .. .. .. 92
5. Effusion of Blood into the Abdomen . .. .. .. .. .. .. .. .. 95
6. Artificial Anus .. .. .. .. .. .. .. .. .. .. .. .. .. .. .. 95
7. Wounds of the other Viscera.. .. .. .. .. .. .. .. .. .. .. 95
8. Treatment of Injuries of the Abdomen.. .. .. .. .. .. .. .. 97

VIII.

Thoughts on the Nature and Treatment of several severe Diseases of the Human Body. By Edw. J. Seymour, M.D. F.R.S. &c. .. .. .. .. .. .. .. 97
1. Pain in the Stomach—Causes and Treatment .. .. .. .. .. .. 98
2. Vomiting, its Causes and Mode of Treatment .. .. .. .. .. .. 100
3. Gout, its Causes and Treatment .. .. .. .. .. .. .. .. .. .. 102
4. Psoriasis and Pompholyx in Gouty Habits .. .. .. .. .. .. .. 105
5. On the Treatment of Sciatica.. .. .. .. .. .. .. .. .. .. .. 106
6. Utility of Opiates in some Forms of Insanity .. .. .. .. .. .. 109
7. Connexion between Melancholia and Phthisis .. .. .. .. .. .. 113

IX.

Transactions of the Provincial Medical and Surgical Association. Vol. XV. .. 114
1. An Experimental Inquiry into the Effects of Hydrocyanic Acid, produced upon Animal Life. By Thomas Nunneley, Esq. F.R.C.S.E... 114
2. Observations on the Operation of Ovariotomy. By George Southam, Esq .. .. .. .. .. .. .. .. .. .. .. .. .. .. .. .. .. .. 121
3. Observations on the Pathology of Abscess of the Heart. By T. H. Stallard, Esq... .. .. .. .. .. .. .. .. .. .. .. .. .. .. .. 122
4. An Essay, Literary and Practical, on Inversio-Uteri. By John Green Crosse, Esq. .. .. .. .. .. .. .. .. .. .. .. .. .. .. .. 124

X.

I. The Construction and Government of Lunatic Asylums and Hospitals for the Insane. By John Conolly, M.D.
II. Journal of Insanity. Vol. III. Utica, U.S.
III. Third Annual Report of the Managers of the State Lunatic Asylum. By Amariah Brigham, M.D. Albany, U.S.
IV. Twenty-second Report of the Officers of the Retreat for the Insane at Hartford, Conn. U. S.
V. Report of the Medical Officers of the Lunatic Asylum for the County of Lancaster .. 128

1. Site and Construction of Asylums .. 129

2. Paucity of In-door Employments .. 131

3. Importance of Educated Attendants .. 132

4. Religious Services—Instruction .. 134

XI.

A Treatise on Fractures in the Vicinity of Joints, and on certain Forms of Accidental and Congenital Dislocations. By Robt. Wm. Smith, M.D. M.R.I.A., &c. 137

1. Fracture of the Neck of the Femur .. 137
2. Fracture of the Lower-end of the Radius .. 143
3. Fractures of the Humerus .. 147
4. Congenital Dislocation of the Shoulder .. 149
5. Dislocations of the Lower Jaw .. 150

XII.

Lectures on the Comparative Anatomy of the Vertebrate Animals. By Richard Owen, F.R.S. &c. .. 152

1. Theory of the Cranial Vertebræ .. 153
2. Teleology of the Skeleton in Fishes .. 166

XIII.

Lettres de Gui Patin. Par J. H. Reveille-Parise, Docteur en Medecine, &c. .. 169

XIV.

I. Dictionary of Practical Medicine. By James Copland, M.D. F.R.S. &c. Art. Hæmagastric Pestilence
II. Report of a Special Committee of the House of Assembly of the State of New York, on the present Quarantine Laws
III. Report of the Fever at Boa Vista. By Dr. Mc William
IV. Letter addressed by Sir William Pym to the Lords of the Council, relative to a Report on the Fever of Boa Vista, by Dr. Mc William, R.N. .. 183

1. The History of the Yellow Fever surrounded with difficulties .. 185
2. Attempts to define it unsatisfactory .. 187
3. Its resemblance to other Intertropical Fevers .. 189
4. Nature and Source of the Black Vomit .. 192
5. Description of Malignant Remittent Fever .. 194
6. Malignant Remittent in Batavia .. 197
7. Dr. Copland's Views of the Ætiology of Yellow Fever .. 202
8. Is Yellow Fever essentially Infectious? .. 205
9. Epidemic Visitations at Sierra Leone and Gibraltar .. 209
10. Influence of Sanitary Improvements at New York .. 212
11. Comparative immunity of Boston .. 215
12. The Fever on board the "Eclair." .. 217
13. The Yellow Fever as it appeared at Boa Vista .. 219
14. Sir Wm. Pym's Observations on Dr. Mc William's Report .. 230
15. Quarantine and other preventive Measures .. 233

XV.

Mental Dynamics, or Groundwork of a Professional Education. By Joseph Henry Green, F.R.S. &c. .. 235

XVI.

The Nature and Faculties of the Sympathetic Nerve. By JOSEPH SWAN .. .. 237
1. Structure and Functions of the Sympathetic .. .. .. .. .. 238
2. Comparative Anatomy .. .. .. .. .. .. .. .. .. .. .. .. .. 239

XVII.

Household Surgery, or Hints on Emergencies. By JOHN F. SOUTH .. .. .. 240

XVIII.

Speech of Lieut.-Gen. Sir Howard Douglas, Bart. M.P. on the Army Surgeon's Bill, 244

XIX.

I. Inhalation of Ether. By J. MASON WARREN, M.D. &c.. .. .. .. .. ..
II. Practical Remarks on the Inhalation of the Vapour of Sulphuric Ether. By W. PHILPOTT BROOKES, M.D. &c. .. .. .. .. .. .. .. .. .. .. .. 246

XX.

Practical Observations on the Pathology and Treatment of certain Diseases of the Skin, &c. By THOMAS HUNT .. .. .. .. .. .. .. .. .. .. .. .. .. 247

XXI.

On Sir Charles Bell's Researches in the Nervous System. By ALEX. SHAW.. .. 249

XXII.

Medical Statistics, their Force and Fallacy. By JAMES DUNCAN, A.M. M.D. .. 250

XXIII.

Remarks on the Diet of Children, &c. By GEORGE T. GREAM .. .. .. .. 251

XXIV.

Gray's Supplement to the Pharmacopœia, &c. By Theophilus Redwood .. .. 283

### PERISCOPE.

### Selections from the Foreign Periodicals.

1. On Letting Blood from the Jugular in the Diseases of Children. By E. Hildreth, M.D. .. .. .. .. .. .. .. .. .. .. .. .. .. .. .. .. .. 252
2. Observations Introductory to a Course of Clinical Surgery. By M. Malgaigne, 253
3. Treatment of Epilepsy .. .. .. .. .. .. .. .. .. .. .. .. .. .. .. 256
4. Treatment of Burns .. .. .. .. .. .. .. .. .. .. .. .. .. .. .. 256
5. Ectrotic Treatment of Small-Pox.. .. .. .. .. .. .. .. .. .. .. 256
6. Rinino on Chronic diffused Arteritis .. .. .. .. .. .. .. .. .. .. 257
7. On Intercostal Neuritis and Neuralgia. By Dr. Beau .. .. .. .. .. .. 259
8. On Exostoses and the Operations they require. By M. Roux .. .. .. .. 262
9. On the Iodide of Potassium in Syphilis. By Dr. Aran .. .. .. .. .. 265
10. On the Use of Assafœtida in Pregnancy. By Dr. Laferla.. .. .. .. .. 266
11. Observations upon the Treatment of Squamous Diseases of the Skin. By M. Devergie.. .. .. .. .. .. .. .. .. .. .. .. .. .. .. .. .. 267
12. Devergie's Observations upon Itch. .. .. .. .. .. .. .. .. .. .. 269
13. A Sketch of the Life and Works of Tommasini. By Prof. Giacomini .. .. 271
14. Practical Observations upon Foreign Bodies introduced into the Eye. By M. Petrequin .. .. .. .. .. .. .. .. .. .. .. .. .. .. .. .. 274
15. On the Dentition of Children. By M. Trousseau .. .. .. .. .. .. .. 275
16. Albuminuria in Pregnant Women .. .. .. .. .. .. .. .. .. .. .. 277
17. Hygienic Rules in Myopia. By Dr. Sichel.. .. .. .. .. .. .. .. .. 278
18. Tanchou on the Indications for Bleeding .. .. .. .. .. .. .. .. .. 279
19. The Pneumonia of Old Persons .. .. .. .. .. .. .. .. .. .. .. .. 281
20. Anesthesia in Saturnine Affections .. .. .. .. .. .. .. .. .. .. 282
21. Alum as an Emetic in Croup .. .. .. .. .. .. .. .. .. .. .. .. 282
21. Cod-Liver Oil.. .. .. .. .. .. .. .. .. .. .. .. .. .. .. .. .. 282
Bibliographical Record .. .. .. .. .. .. .. .. .. .. .. .. .. .. 283

